# Post mortem computed tomography meets radiomics: a case series on fractal analysis of post mortem changes in the brain

**DOI:** 10.1007/s00414-022-02801-5

**Published:** 2022-03-03

**Authors:** Fabio De-Giorgio, Gabriele Ciasca, Gennaro Fecondo, Alberto Mazzini, Riccardo Di Santo, Marco De Spirito, Vincenzo L. Pascali

**Affiliations:** 1 Fondazione IRCCS Policlinico Universitario A. Gemelli, Rome, Italy; 2 Section of Legal Medicine, Department of Healthcare Surveillance and Bioethics, Università Cattolica del Sacro Cuore, Rome, Italy; 3 Department of Neuroscience, Section of Physics, Università Cattolica del Sacro Cuore, Rome, Italy

**Keywords:** Post-mortem changes, Post-mortem interval, Computed tomography, Radiomics, Virtopsy, Fractal analysis, Image analysis, Quantitative imaging

## Abstract

Estimating the post-mortem interval is a fundamental, albeit challenging task in forensic sciences. To this aim, forensic practitioners need to assess post-mortem changes through a plethora of different methods, most of which are inherently qualitative, thus providing broad time intervals rather than precise determinations. This challenging problem is further complicated by the influence of environmental factors, which modify the temporal dynamics of post-mortem changes, sometimes in a rather unpredictable fashion. In this context, the search for quantitative and objective descriptors of post-mortem changes is highly demanded. In this study, we used computed tomography (CT) to assess the post-mortem anatomical modifications occurring in the time interval 0–4 days after death in the brain of four corpses. Our results show that fractal analysis of CT brain slices provides a set of quantitative descriptors able to map post-mortem changes over time throughout the whole brain. Although incapable of producing a direct estimation of the PMI, these descriptors could be used in combination with other more established methods to improve the accuracy and reliability of PMI determination.

## Introduction

The notion of fractal geometry was first introduced by Benoit B. Mandelbrot and has nowadays found application in multiple fields of study. Fractals are defined as “a fragmented geometry whose subdivisions are approximates of the whole geometry” and can commonly be found in nature (i.e., shorelines, clouds, snowflakes, molecular structures, vascular trees) [1, 2]. Thus, with the term “fractal geometry,” we refer to complex systems that display particular patterns which cannot be easily described using the traditional Euclidian methods [3]. When applied to clinical and research settings, fractal geometry (fractal analysis) allows quantifying texture (heterogeneity) on digital images with the use of mathematical models [4–6]. In this regard, several studies concerning the combined use of fractal analysis and computed tomography (CT) for clinical purposes can be found in the literature. For instance, this method was used to assess the characteristics of brain and liver tumors [7, 8], brain lesions [9], lung nodules [10], lymph node metastases [11, 12], and lacunarity degree in Alzheimer’s disease [13].

In particular, by virtue of its peculiar anatomical structure and inaccessibility, the brain represents an ideal candidate for this new field of research, both ante- and post-mortem. There are studies in the literature that discuss the examination of the post-mortem brain as a means of determining the PMI; however, these focus on methodologies other than fractal analysis. For instance, Schmidt et al. [14] examined 21 subjects to investigate the temporal pattern of the apparent diffusion coefficient in the brain (thalamus, brain, and cerebellum) after death. The study’s findings revealed a distinct pattern during the examination time, with substantial variations among ADCs ex vivo and in vivo. Musshoff et al. [15] and Ith et al. [16], on the other hand, used in situ proton magnetic resonance spectroscopy (1H-MRS) to study the time-dependent metabolic changes in post-mortem sheep brain and selected human cases for PMI estimation, with promising results. With regard to fractal analysis, Jauhari et al. [9] used the theory of fractal geometry to analyze and quantify digital CT images of the human brain. According to the authors, the fractal theory represents a useful tool for the characterization and diagnosis of CT images, which may help differentiate between normal and diseased brain CT findings. In a study by Iftekharuddin et al. [17], the authors analyzed the potential applications of fractal-based algorithms in terms of brain tumor identification on magnetic resonance (MR) images. The fractal dimension allowed the authors to detect brain tumors when reference non-tumor images were available. A continuation of this study was carried out by Zook et al. [8] who investigated the results of fractal dimension analysis on a set of MR and CT images for statistical validation, eliminating the need for reference images.

Concerning forensic science, to the best of our knowledge, there are no studies concerning the application of fractals to post-mortem brain CT images [18]. In particular, we were not able to find reports regarding the use of this technique for the determination of the time of death (or post-mortem interval, PMI). Thus, the purpose of this study was to apply the concept of fractal analysis to the CT evolution of normal post-mortem changes, to identify patterns that could help in the determination of the PMI.

## Materials and methods

### Subject recruitment and CT measurements

**Fig. 1:**
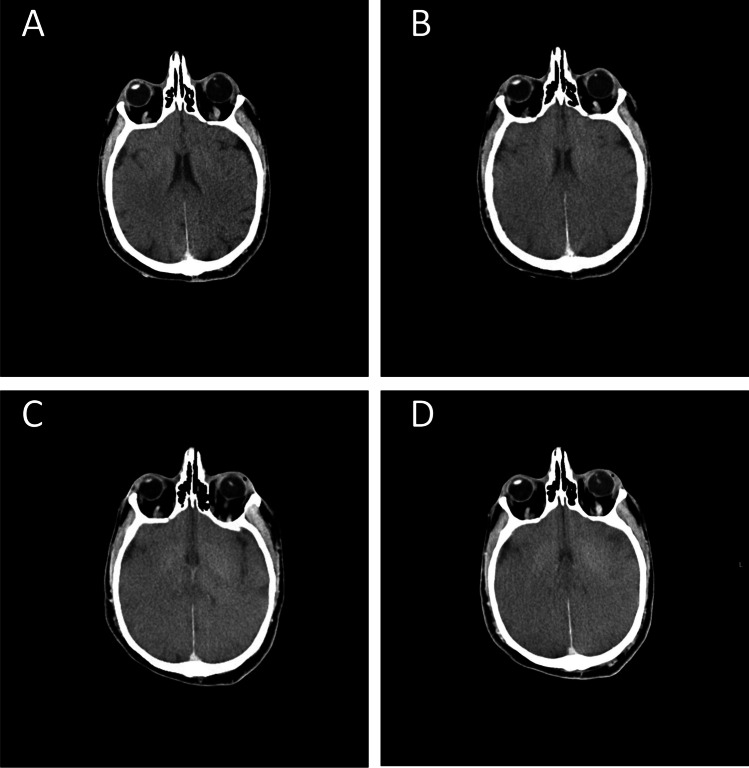
Representative CT brain slices acquired on one of the subjects included in the study at different PMT, namely day 1 (**A**), day 2 (**B**), day 3 (**C**), and day 4 (**D**)

A total of four corpses with a mean age at the time of death of 53.7 years (SD = 11.61) and an average BMI of 20.9 (SD = 1.65) were included in the study. All subjects were males (Table 1). Inclusion criteria were a defined time point of death that occurred during the day and with witnesses (as reported by medical doctors) and age above 18 years. Criteria used to exclude brains from the study were the presence of cerebral organic diseases and/or other traumatic damages. Before autopsy, each corpse underwent consecutive PMCT scans. The bodies were placed on a horizontal CT table in the supine position with their arms at their sides. They were fully clothed and wrapped in body bags. The exams were conducted with the use of a Somatom Scope 16-slice CT scanner (Siemens Healthineers, Italia) using head district protocol equipped with H31S kernel, at a nominal beam energy of 130 kVp and workload of 150 mAs. Images for 512 × 512 pixels were acquired with a 2.4-mm slice thickness -> 512 × 512 pixel images were acquired with a 2.4-mm slice thickness. Both cranial and whole-body CT scans (from the skull vertex to the most distal point allowable, up to about 2000 mm) were obtained. No contrast agent was used in this procedure. Two sets of scans were performed: one from the skull vertex to the sternal notch (1-mm reconstructions), and one from the skull vertex to beyond the feet (1.5-mm reconstructions). Scans were repeated if artifacts were present. In each case, the first PMCT examination was performed at 24-h post-mortem, and the last at 96-h post-mortem; images were acquired every 24 h for 4 days, yielding a total of 4 PMCT scans per body. This technique of consecutive PMCT scans technique allowed us to compare images of the same structures at different post-mortem times. Four representative CT brain slices acquired at different times on one of the recruited corpses are reported in Fig. 1A–B.

**Table 1:** Detailed list of the studied cases, provided with baseline parameters (gender, age), cause of death, and time interval between the first and the last PMCT scans

Case	Gender	Age	Cause of death	First PMCT scan (hours)	Last PMCT scan (hours)
A	M	39	Hanging	24	96
B	M	46	Sudden cardiac death	24	96
C	M	67	Sudden cardiac death	24	96
D	M	63	Hanging	24	96

The cadavers were kept in the same position for the entire procedure (from the first scan session to the last) to provide reliable and reproducible results. The temperature in the CT room was maintained at 18 °C and air humidity was set at 49%. Throughout the examination, the bodies were kept in said room. All corpses had a rectal temperature of 18 °C at the time of the initial PMCT assessment; this temperature was maintained throughout the examination period. Following the PMCT examination, the corpses were autopsied, and histological/toxicological analyses were performed.

### Image and statistical analysis

**Fig. 2:**
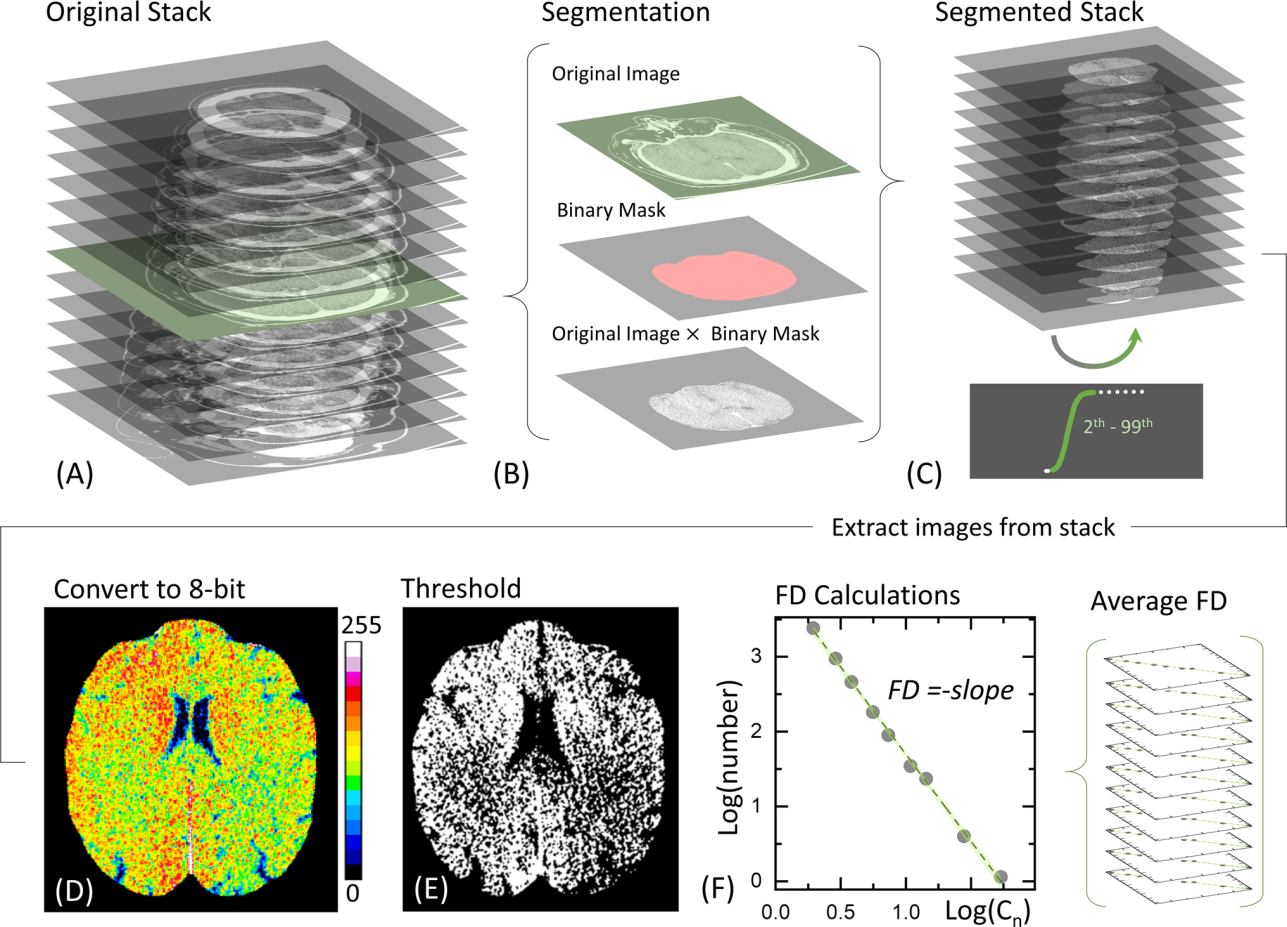
Schematic visualization of the image analysis steps. Representative image stack (**A**). Image segmentation (**B**). Segmented stack and outlier removal (**C**). Representative segmented CT slice obtained after 8-bit conversion (**D**). Same image at the 0.55 threshold level (**E**). Box-counting plot for calculating FD (**F**) and average over the whole stack

Images of the four analyzed corpses were acquired with the use of a Siemens Sensation CT scanner. Brain region slices were extracted and stored in a single image stack for subsequent image processing. Slice selection was performed according to the anatomical structure, considering the entire intracranial region (Fig. 2A). Image segmentation was performed with the ImageJ *Segmentation Editor* internal plugin, as described in the following (Fig. 2B). A first contoured region was automatically selected using the magic wand tool, setting Hounsfield units from − 5 to 65, according to [19, 20]. Manual refinement of each contoured region was then performed. Thinking of a potential practical application of the proposed technique, it is worth commenting on the time required for image analysis. A time interval ranging between 1 and a few hours can be estimated for the segmentation of each brain stack. The resulting slices were binarized, stored in an image stack, and used as a mask to isolate the brain radiodensity maps used in the subsequent analysis. In this step, the original stack was multiplied by the mask (Fig. 2B), and the resulting pixel values of global histogram was equalized after removing pixels of the 1st and the 99th percentiles (Fig. 2C), and then transforming it to an 8-bit image sequence (Fig. 2D), as suggested in [21]. The obtained stack was then processed with a homemade ImageJ macro developed to calculate the fractal dimension (FD) after image thresholding (Fig. 2E) for each image using the box-counting method implemented in ImageJ (Fig. 2D), according to [22]. The ImageJ internal tool adopted applies covering squares—with side values of 2, 3, 4, 6, 8, 12, 16, 32, and 64 pixels respectively—to cover the whole image, and then it plots the log–log graph of the minimum number of squares required to cover the edges of the binarized image (referred to as “number” in Fig. 2) versus the side value adopted (referred to as “Cn” in Fig. 2). At variance with the first segmentation steps, this phase of the analysis can be conducted almost in real-time through the application of the abovementioned ImageJ macro. All the image analysis steps carried out so far have been schematically summarized in Fig. 2A–F.

**Fig. 3:**
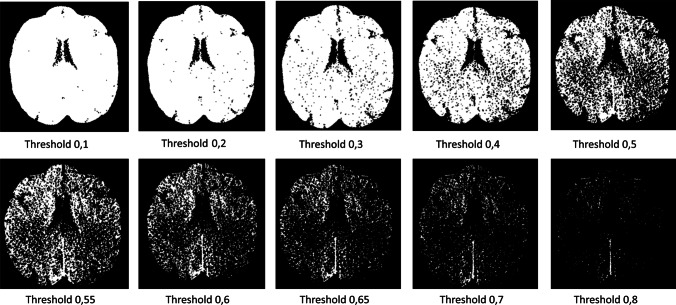
Different binarization outputs as a function of the threshold values adopted. For example, a threshold value of 0.2 means that the 1 − 0.2 = 80% of the histogram is set to 1 (white)

FD computed with this method strongly depends on the threshold value used for image binarization. Therefore, we tested FD at different threshold levels ranging from 0.1 to 0.8 at increasing steps of 0.05. For each subject, each threshold, and each time point, FD is reported in terms of mean ± SEM, computed over the entire stack (a representative image at different thresholds in the investigated range is reported in Fig. 3).

## Results

In this section, we investigate whether fractal analysis of CT brain slices can provide a set of quantitative descriptors (i.e., morphological modifications over time such as a progressive loss of brain convolutions and ventricular volume) capable of mapping post-mortem alterations overtime throughout the whole brain.

In Fig. 1, we depict the time evolution over 4 days of four representative CT slices acquired on one of the subjects included in the study. A visual examination of Fig. 1 reveals a progressive loss of brain convolutions over time, which is likely associated with a change in the perimeter/area ratio of the selected anatomical region. We employed the fractal dimension (FD), which can be derived for each slice using the box-counting method implemented in ImageJ after slice segmentation and binarization, to quantitatively monitor these changes over time. The exploited image analysis algorithm is described in material and methods and summarized in Fig. 2. Briefly, relevant slices were selected (Fig. 2A); each slice was segmented creating a binary mask and then applying it to the original image (Fig. 2B). Outliers were removed filtering HU values lying outside the 1st–99th percentile range computed over the entire segmented stack (Fig. 2C). Subsequently, segmented slices were converted to 8-bit format (Fig. 2D) and a threshold was applied. Figure 2E shows a representative image, where 55% of the analyzed pixels are set to white and the rest is set to black (0.55 threshold level). Fractal dimension (FD) was computed for each slice using the box-counting method implemented in ImageJ and then averaging the values obtained on the entire stack (Fig. 2F). Figure 2F shows a representative box-counting plot, which displays the log–log graph of the minimum number of squares required to cover the edges of the binarized image (Log(*number*)) versus the side value of the same squares (log(Cn)). In this representation, FD corresponds to the modulus of the slope in the linear fit (Fig. 2F). The best regression line is reported together with the corresponding 95% CI.

A major concern regarding the FD parameter is the fact that it is strongly related to the threshold level set for the analyzed images [17]. To account for this dependency, we decided to compute the average FD at different threshold levels from 0.1 to 0.8 (Fig. 3) and study its evolution as a function of the threshold. Moreover, as a single slice is not representative of the whole brain behavior; we applied this method to each brain slice of each subject at a given time point, and we computed the average FD value at each threshold level.

**Fig. 4:**
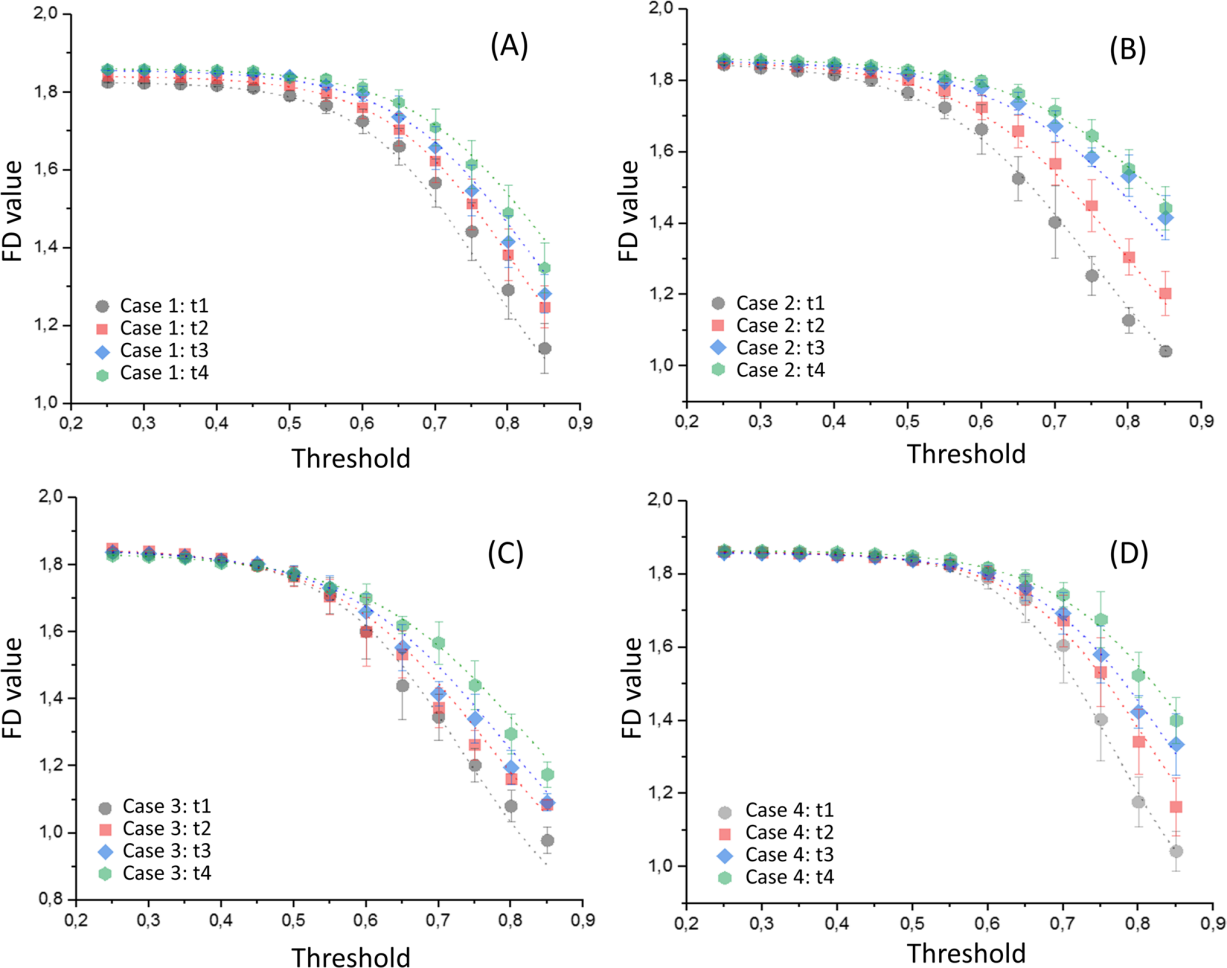
Plot of FD data as a function of the threshold value, for day 1 (gray circle), day 2 (red square), day 3 (blue turbot), and day 4 (green hexagon) for the whole dataset available. Cases 1, 2, 3, and 4 are represented in panels **A**, **B**, **C**, and **D**, respectively. A Boltzmann sigmoidal function is fitted to each experimental trend

**Fig. 5:**
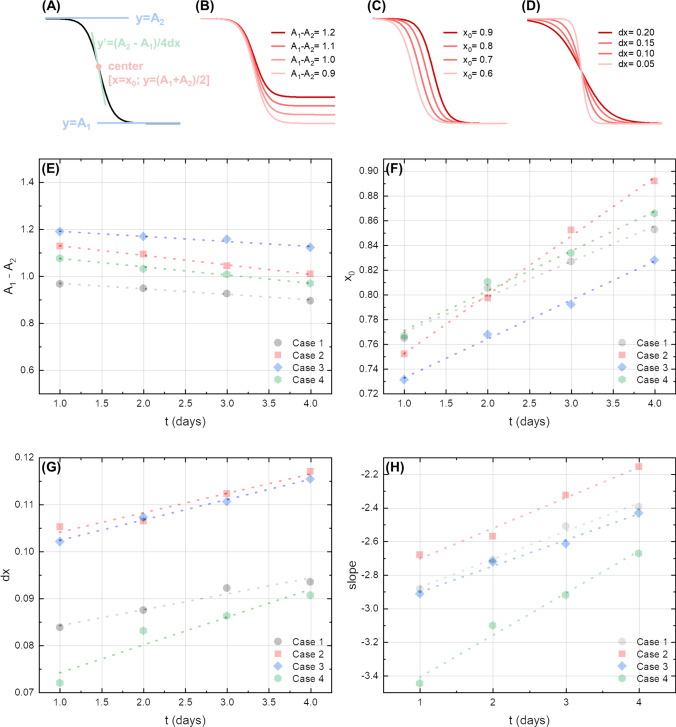
Graphical visualization of the meaning of the fitting parameters of the Boltzmann function using a simulated curve (**A**). Simulated data showing how the Boltzmann function changes as a function of the span (**B**), the center *x*_0_ (**C**), and the constant *dx* (**D**). Scatter plots of the four main fitting parameters retrieved from the sigmoidal fit of the data in Fig. 4, namely: the span, A_1_ − A_2_ (**E**), the center, *x*_0_ (**F**), the constant, *dx* (**G**), and the slope (**H**). Different subjects are represented with different colors and symbols

In Fig. 4, we show the behavior of the FD value averaged over the whole image stack as a function of the threshold level for all the cases recruited in the study, namely case 1 (Fig. 4A), case 2 (Fig. 4B), case 3 (Fig. 4C), and case 4 (Fig. 4D). Different curves were plotted at different times after death for each subject, from day 1 to day 4. One can notice that each of the computed curves shows a similar behavior, starting from a plateau at low threshold values and then decreasing monotonously with increasing thresholds. This qualitative behavior is highly conserved, independently of the subjects and particular time-point. Looking at the FD vs threshold curves at different times, a monotonous shortening of the initial plateau region can be observed going from day 1 to day 4. We quantified this behavior fitting all the acquired curves with the sigmoidal Boltzmann function $FD={A}_{2}+\frac{\left({A}_{1}-{A}_{2}\right)}{1+{e}^{x-{x}_{0}/dx}}$, where *x* is the threshold level, *A*_1_, *A*_2_, *x*_0_, and *dx* are fitting parameters. A good agreement between the phenomenological model (dotted line) and the experimental points can be observed for each data-set, with *R*^2^ values close to 1. In Fig. 5, we investigate the possibility of using the abovementioned fitting parameters as quantitative markers to monitor post-mortem changes. For the sake of clarity, we show graphically how these parameters contribute to determining the shape of the curve using simulated data (Fig. 5A). Similarly, to better clarify the meaning of these parameters, in Fig. 5B–D, we show a set of simulated curves in which one single parameter is varied at a time.

In Fig. 5E–H, we display the behavior of the experimental fitting parameters retrieved from Fig. 4 as a function of the time after death for each subject, namely A_1_ − A_2_ (Fig. 5E), *x*_0_ (Fig. 5F), *dx* (Fig. 5G), and the slope (Fig. 5H). For each of the analyzed subjects, the fitting parameters show a strong inter-independent variability regarding absolute values, but similar time trends over the four analyzed days. Specifically, the baseline difference A_1_ − A_2_ mildly and monotonously decreases over time, while *x*_0_, *dx*, and slope increase over time.

## Discussion

In recent years, CT has been used as a complementary tool to autopsies, gaining increasing importance in the field of forensic science [23–25]. Nonetheless, despite its multiple forensic applications, post-mortem CT has mainly been used as a method to determine the cause of death; this is especially true if we consider the historical period that we are living in. While this does not apply to all forensic institutes, many of them have reduced the number of autopsies during the pandemic for safety reasons; in this scenario, post-mortem CT proved once again useful as both a diagnostic and screening tool [26–28]. Concerning the determination of the PMI, however, only a few studies that focus on the use of post-mortem CT can be found in the literature. Undoubtedly, in the past years, efforts have been made by the forensic community, including our research team, to identify methods that could help determine the time of death, such as the study of human tissues transcriptomes [29], muscle proteins degradation [30], gene expression patterns [31], ocular changes, [32, 33], metabolomics [34–36], the study of rigor mortis using ultrasound shear wave elastography (US SWE) [37] or atomic force microscopy (AFM) [38]. However, to our knowledge, there are no studies concerning the application of fractal analysis to post-mortem CT scans for the determination of the PMI.

Because of its anatomical structure and limited access to it, as it is enclosed in the meninges, the brain represents a very important organ to investigate this type of problem. The appearance of gas in the various structures of the brain, and the loss of differentiation between white and gray matter are generally among the first post-mortem alterations impacting the brain [39–41]. Concerning our experiment, it demonstrates a temporal evolution of the brain morphology which derives from a progressive loss of the ventricular volume [42, 43] and the folded structure of the gray matter. Although these aspects can be qualitatively observed, in forensic sciences, there is a demanding need to establish new quantitative parameters. For this reason, we investigated the temporal variation of the average FD value, calculated on the brains of four subjects whose time of death was known.

The average FD computed on each brain slice displays a coherent trend among subjects, thus showing potential to be used for the quantitative monitoring of post-mortem changes over time. As FD is sensible to the threshold applied to the binarized image, we extended the analysis by studying the FD variation in function of the different threshold levels adopted over time. For each time point, we studied the entire behavior of a curve as a function of the threshold, a more informative approach compared to a single threshold value analysis. In particular, we demonstrated that the FD trend with threshold value is compatible with a decreasing sigmoidal function, which fits all the subject data at all different times. From this function, it is possible to deduce quantitative parameters, which can be associated with brain morphological modifications over time. In particular, preliminary results of this study conducted on four subjects indicate two parameters obtained from the sigmoidal regression which are particularly responsive to the temporal changes.

At present state, this is a preliminary study, since our sample population consists of only four subjects; certainly, this does not allow us to obtain significant results and only allows us to report the observed findings and changes. Thus, while our study opens the door to numerous new research possibilities, future studies should undoubtedly be conducted on a larger number of cases, evaluating a longer time interval with particular reference to the initial phases of the first 24 h, and then extending beyond the limit of 4 days. It is also worth noting that, in terms of imaging modality, MRI offers superior diagnostic capabilities when compared to CT, especially for the analysis of soft tissues and organs such as the brain. As a result, future research should consider employing this imaging technique. About the obtained results, the values that were determined are not sufficient to trace back to the time of death; however, we have demonstrated that, by using this methodology, one can obtain a set of valid quantitative parameters that, in more detailed studies concerning the early and late stages of a corpse’s degradation process, could be related to the time of death.
